# Preparation of Azithromycin Amorphous Solid Dispersion by Hot-Melt Extrusion: An Advantageous Technology with Taste Masking and Solubilization Effects

**DOI:** 10.3390/polym14030495

**Published:** 2022-01-26

**Authors:** Jiale Li, Conghui Li, Hui Zhang, Xiang Gao, Ting Wang, Zengming Wang, Aiping Zheng

**Affiliations:** 1 School of Pharmacy, Anhui Medical University, 81th Meishan Road, Hefei 230032, China; lijiale253@163.com; 2 State Key Laboratory of Toxicology and Medical Countermeasures, Beijing Institute of Pharmacology and Toxicology, 27th Taiping Road, Haidian District, Beijing 100850, China; angelina52819@163.com (C.L.); zhhui58@126.com (H.Z.); gaoxiang609@163.com (X.G.)

**Keywords:** pediatric preparation, hot-melt extrusion, azithromycin, polymer, amorphous solid dispersion, design of experiment

## Abstract

Azithromycin (AZI) is one of the most commonly used macrolide antibiotics in children, but has the disadvantages of a heavy bitter taste and poor solubility. In order to solve these problems, hot-melt extrusion (HME) was used to prepare azithromycin amorphous solid dispersion. Preliminary selection of a polymer for HME was conducted by calculating Hansen solubility parameter to predict the miscibility of the drug and polymer. Eudragit^®^ RL PO was chosen as the polymer due to its combination of taste-masking effect and dissolution. Moreover, the solubility was improved with this polymer. Design of experiments (DoE) was used to optimize the formulation and process, with screw speed, extrusion temperature, and drug percentage as independent variables, and content, dissolution, and extrudates diameter as dependent variables. The optimal extrusion parameters were obtained as follows: temperature—150 °C; screw speed—75 rpm; and drug percentage—25%. Differential scanning calorimetry (DSC) and Powder X-ray Diffraction (PXRD) studies of the powdered solid dispersions showed that the crystalline AZI transformed into the amorphous form. Fourier transform infrared spectroscopy (FTIR) results indicated that the formation of a hydrogen bond between AZI and the polymer led to the stabilization of AZI in its amorphous form. In conclusion, this work illustrated the importance of HME for the preparation of amorphous solid dispersion of AZI, which can solve the problems of bitterness and low solubility. It is also of great significance for the development of compliant pediatric AZI preparation.

## 1. Introduction

Nearly 7 million children under the age of 5 die every year, usually from treatable diseases [1]. The main reason is the lack of adequate and regular pediatric preparations [1,2]. A drug suitable for children is a prerequisite for the treatment of childhood diseases, especially the taste of the drug, which largely affects the acceptance of pediatric patients and thus the effectiveness of the drug [1,3,4]. It is meaningless to conduct clinical trials of products with an unpleasant taste in the pediatric population. Therefore, special attention should be paid to the bitterness of drugs in the development of pediatric preparation [1,5].

Azithromycin (AZI) is one of the world’s best-selling broad-spectrum macrolide antibiotics and the most commonly used antibiotics in children [6]. However, AZI has a heavy bitter taste, which leads to poor oral compliance in children [7]. Likewise, AZI has poor water solubility and low dissolution rate in the gastrointestinal tract, which limits the bioavailability after oral administration [8,9]. The simplest and most frequently used method to mask the taste of pediatric formulations is to add flavors and sweeteners, but this is not feasible for AZI [7]. Some studies have shown the use of physisorption technique [7], ion exchange resin technique [10], and reverse micelles technique [11] to mask the bitterness of AZI. However, these techniques are operationally complex and, therefore, there is a necessity for a simpler approach to develop more compliant AZI preparation for children.

Solid dispersion (SD) has been widely developed as a formulation technology to improve drug solubility, increase oral bioavailability and mask taste [12,13]. It refers to one or more active pharmaceutical ingredients (API) dispersed in an inert polymer or matrix. The mechanism mainly includes: particle size reduction, amorphous API, high porosity and high wettability [12,14,15,16]. The preparation methods mainly include solvent methods and melt methods. Hot-melt extrusion (HME) technology is one of the common technologies used for the preparation of SD by melt method.

With the development of drugs with low solubility and poor bioavailability, HME is gradually being applied to the field of pharmaceutical preparations [17]. HME is a semi-continuous or continuous preparation process that includes melting, mixing, homogenizing, and extrusion. It has the advantages of fewer processing steps, solvent-free process, continuous operation, easy online process analysis, and easy amplification. HME has emerged as an alternative platform technology to other traditional techniques for manufacturing pharmaceutical dosage forms, such as implants and transdermal and transmucosal drug delivery formulations [17,18,19,20]. HME provides energy through heating and mechanical shear to make the crystalline API transform into amorphous state. At the same time, in the molten state, the particle size of the material continuously decreases, and the amorphous API exchanges and penetrates with the polymer to form solid solution (SS). The products produced by HME generally have dense particles, suitable fluidity and small differences between batches, and are commonly used in commercial production. Therefore, HME technology was chosen to prepare AZI solid dispersions.

In this study, Eudragit^®^ RL PO was innovatively used as a polymer to prepare solid dispersions by HME using AZI as a model drug to solve the problems of bitterness and solubility. At the same time, the optimal preparation process parameters of HME and physicochemical properties of SD were also studied. The results demonstrate that the combined application of Eudragit^®^ RL PO and HME is a good solution to the above problems, while the AZI is converted from the crystalline to the amorphous state. The advantages of the amorphous state, the method of production and the SD have been harnessed to produce formulations by synergizing the benefits. This also provides significant advantages for the development of compliant pediatric AZI preparation.

## 2. Materials and Methods

### 2.1. Materials 

AZI was purchased from CSPC (Shijiazhuang, China). Eudragit^®^ RL PO and Eudragit^®^ RS PO were received as samples were from Evonik (Frankfurt, Germany). Compritol^®^ 888 ATO (Glyceryl behenate, GB) and Klucel™ HPC LXF was donated by the GATTEFOSSÉ (Lyon, France) and Ashland (Covington, KY, USA). Affinisol™ HME 15LV (Hydroxypropyl Methylcellulose, HPMC 15LV) and Kollidon^®^ K30 (Polyvinyl pyrrolidone, PVP K30) were kindly gifted by Dow (Midland, MI, USA) and BASF (Ludwigshafen, Germany). All solvents were of analytical grade.

### 2.2. Hansen Solubility Parameter (HSP)

HSP was proposed by Hansen in 1967 to predict the miscibility between different material systems. In HME technology, it is possible to judge whether the carrier and API are compatible. Generally, when the difference of HSP is less than 7 MPa^0.5^, it indicates that the drug and the polymer may have good compatibility [21].

HSP originated from the Hildebrand solubility parameter. The Hildebrand solubility parameter uses a single Cohesive Energy Density (CED) value to predict the miscibility of two substances, which is not suitable for strong polar interactions. HSP refines the types of molecular interactions based on the Hildebrand solubility parameter, and considers three forces that affect solubility, namely polar, dispersion, and hydrogen bonding force. HSP is expressed as “*δ*”, and its international standard unit is “MPa”. Based on the different groups with different contributing components, the HSP of the compound is calculated by the group contribution method, the calculation formula is as follows [18,22,23,24]:

(1) $$\delta^{2}=\delta_{d}^{2}+\delta_{p}^{2}+\delta_{h}^{2}$$

(2) $$\delta_{d}=\frac{\sum^{\;}F_{di}}{V}\;\delta_{p}=\frac{\sqrt{\sum^{\;}F_{pi}^{2}}}{V}\;\delta_{h}=\sqrt{\frac{\sum^{\;}E_{hi}}{V}}$$

Among them, “*δ*” is the HSP, “*δ_d_*” is the dispersive force component, “*δ_p_*” is the polar force component, “*δ_h_*” is the hydrogen bonding force component, “*F_di_*” is the contribution of the dispersive force group, “*F_pi_*” is the polar force group contribution, “*E_hi_*” is the contribution of the hydrogen bonding group, and “*V*” is the molar volume.

The HSP of azithromycin is calculated by the group contribution method, and the HSP of some polymers is taken from the literature.

### 2.3. Preparation of Solid Dispersion by HME

**Figure 1:**
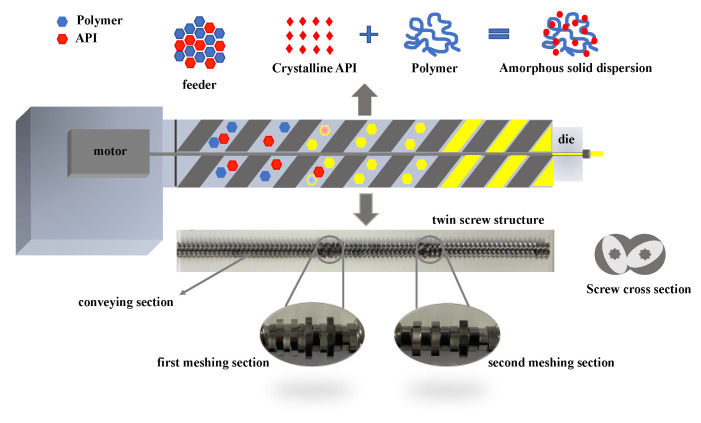
Co-rotating twin screw melt extruder.

The drug and the polymer materials were homogeneously mixed to obtain a physical mixture (PM). SD was prepared using a co-rotating twin screw melt extruder (Pharma11 Melt extruder from Thermo Scientific, Karlsruhe, Germany). When the extruder reached the preset temperature, we turned on the low screw speed, and manually fed about 30–40 g of PM into the feed port. After the material was extruded, the screw speed was increased. The extrusion temperature needed to be set according to the properties of the AZI and the polymer, and the temperature was generally increased with a stepped section of 5–10 °C. The twin screw is the core component of the HME technology, which is mainly composed of a conveying section and a meshing section. This study used a two-stage meshing section screw design, as shown in Figure 1.

A cylindrical extrusion die with a diameter of 2 mm was used to extrude the molten material, and a 485 mm conveyor belt (Pharma 11 conveyor from Thermo Scientific, Karlsruhe, Germany) was used to cool and collect the extrudate. The extrudate was pelletized by a pelletizer (varicut pelletizer 11 MM from Thermo Scientific, Karlsruhe, Germany) at a high speed and then sieved to obtain a powdery solid dispersion.

### 2.4. High Performance Liquid Chromatography (HPLC) Analysis

The analysis of the drug was performed using an U3000 Thermo HPLC (Thermo Scientific, Karlsruhe, Germany). Using Welch Xtimate C18 (4.6 mm × 250 mm, 5 μm) chromatographic column, the mobile phase was a mixture of 35% 4.6 g/L anhydrous potassium dihydrogen phosphate buffer salt solution (pH7.5) and 65% acetonitrile. Flow rate was 1.2 mL/min, sample volume was 50 μL, column temperature was 50 °C, and the UV detection wavelength was 210 nm. The samples were analyzed with the mobile phase as solvent at a concentration of 1 mg /mL, filtered and injected directly into the column.

### 2.5. Dissolution Testing

Dissolution studies were performed in a USP II paddle dissolution apparatus (RC1207DP Automatic dissolution apparatus, Tianda Tianfa Technology Co., Ltd., Tianjin, China). The dissolution medium was 900 mL of phosphate buffer (pH 6.0), the speed was 100 rpm and the temperature was 37 °C. Powdered solid dispersion was added to the dissolution tank. A total of 5 mL of the sample was withdrawn at 60 min, syringe filtered and analyzed by High Performance Liquid Chromatography (HPLC) as described above.

### 2.6. Saturation Solubility Testing

The solubility of the sample was determined by the shake flask method. The excess sample was dissolved in 25 mL distilled water in a 50 mL centrifuge tube and placed in a constant temperature shaker at 37 °C and shaken at 200 rpm for 24 h (HYN-200D Constant temperature culture oscillator, Tianjin Honour Instrument Co., Ltd., Tianjin, China). The supernatant was taken and filtered by syringe, then analyzed by HPLC as described above.

### 2.7. Physicochemical Characterization of the Extrudate

#### 2.7.1. Thermogravimetric Analysis (TGA) 

In order to study the thermal stability of materials, thermogravimetric analysis (TGA) was performed using a TGA Q50 (TA Instruments, New Castle, DE, USA). An appropriate amount of sample was taken in an aluminum sample pan, maintained in a dry nitrogen environment (60 mL/min), and heated from 30 °C to 500 °C at a rate of 10 °C/min. The weight loss samples were recorded.

#### 2.7.2. Differential Scanning Calorimetry (DSC)

A TA Instruments Q2000 DSC equipped with a refrigerated cooling system (TA Instruments, New Castle, DE, USA) was used to record the thermogram. The enthalpic response was calibrated with Indium and zinc. An empty sealed pan was used as a reference. An appropriate amount of the sample was taken in an aluminum sample tray and kept in a dry nitrogen environment (50 mL/min). After the system environment was stabilized, the temperature was increased from 30 °C to 210 °C at 10 °C/min and the thermogram of the sample was recorded. 

#### 2.7.3. Powder X-ray Diffraction (PXRD) 

A Bruker D2 PHASER (Bruker AXS GmbH, Karlsruhe, Germany) X-Ray Diffractometer equipped with a Cu Kα radiator of 30 kV and 10 mA was used. The angular range was 4–50°, the step size was 0.02°, and the speed was 2°/min.

#### 2.7.4. Fourier Transform InfraRed (FTIR) Spectrophotometric Analysis

FTIR was performed on a Nicolet iS5 FT-IR spectrometer (Thermo Scientific, Karlsruhe, Germany). The powder samples were made into KBr pellets and examined by infrared spectroscopy in the 400–4000 cm^−1^ wavenumber range with a resolution of 4 cm^−1^.

### 2.8. Evaluation of Taste Masking Effect

The taste-masking effect evaluation method used the classical population taste evaluation method and was analyzed using the integrated score evaluation method (ISEM). ISEM was tested in a single-blind, randomized manner. After tasting the drug, volunteers ranked the bitterness according to their subjective taste perception and filled in the “drug bitterness ranking score scale”.

Five healthy young people were chosen as subjects (denoted as A, B, C, D, E). They tasted the sample to be tested (40 mg of solid dispersion powder) for 10 s and spat it out, rinsed their mouths with pure water 5 times, and then tested the next batch of samples every 30 min. The bitterness evaluation standard is shown in Table 1. Bitterness is split into 6 levels, each with a certain bitterness score, with scores from 1 to 6 and increasing bitterness in that order.

### 2.9. DoE Design and Implementation

A full factorial experimental design was conducted through preliminary risk assessment and single-factor experimental study. Screw speed, extrusion temperature, and drug percentage were selected as independent variables, and content, dissolution rate, and extrudate diameter were used as dependent variables to study the interaction of independent variables and determine the design space.

The independent variable level and response value range are shown in Table 2, and the running sequence is shown in Table 3. Data were analyzed and plotted using Minitab software (Minitab LLC., University Park, PA, USA) to assess the effects of the respective variables on the dependent variable and the interactions between the variables.

## 3. Results

### 3.1. Polymer Selection

#### 3.1.1. Hansen Solubility Parameter

**Figure 2:**
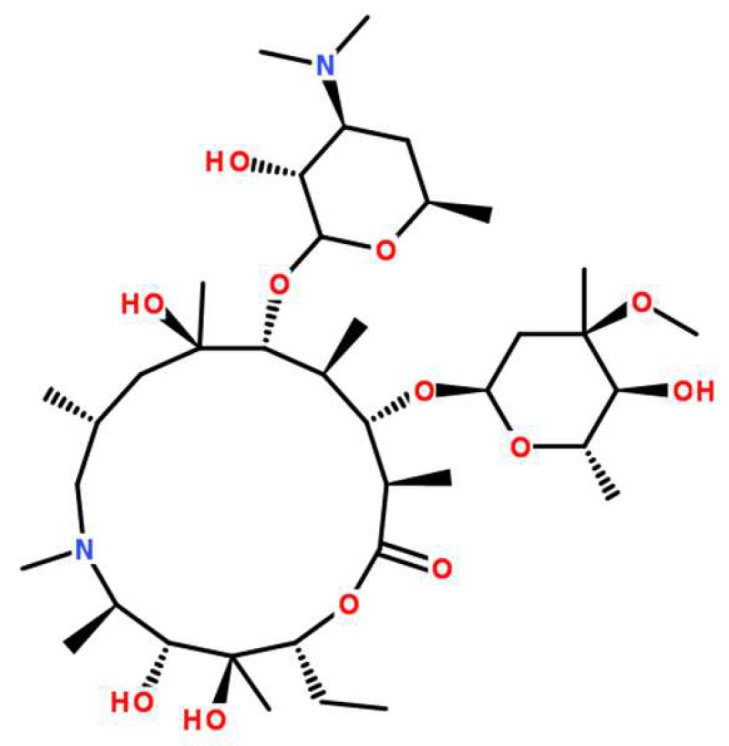
Structural formula of Azithromycin.

The structural formula of AZI is shown in Figure 2. The contribution components of polar force, dispersion force and hydrogen bond force of different groups are shown in Table 4. According to Formulas (1) and (2), the HSP of AZI is calculated to be 25.99 MPa^0.5^.

The selection of the polymer requires consideration of the suitability of the formulation design, mainly in terms of compatibility with API [5]. Therefore, the HSP can be calculated based on the chemical structure to predict the solid–solid miscibility of the drug and the polymer. The HSPs of different polymers were obtained as shown in Table 5. The HSP difference (∆δ) between AZI and Eudragit^®^ RL PO, Eudragit^®^ RS PO, GB, PVP K30, HPC LXF, HPMC 15LV are all less than seven, which indicates their good compatibility with AZI. Therefore, we initially screened these six polymers to prepare AZI-SD.

#### 3.1.2. Thermal Stability of AZI

Hot-melt extrusion uses temperature and shear to process a physical mixture into a solid dispersion. The thermal stability of the drug will play a key role in the selection of extrusion parameters.

**Figure 3:**
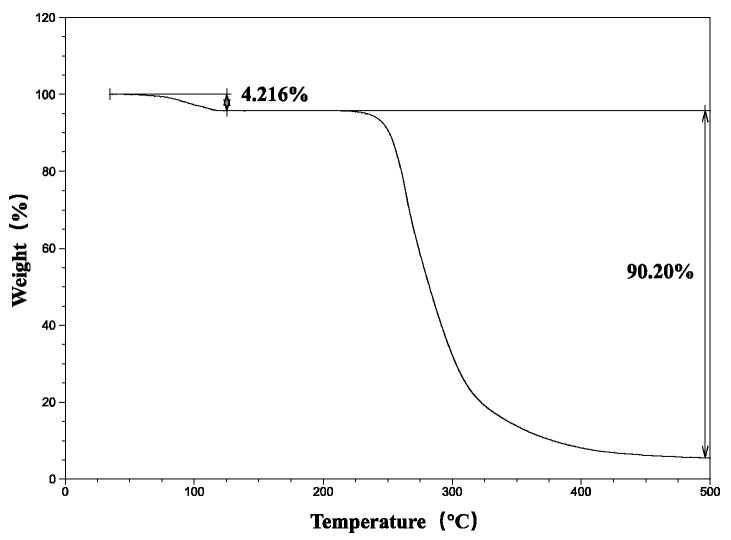
TGA of AZI.

The TGA of the pure crystalline drug shows that AZI begins to decompose above 200 °C, with a weight loss of 4.2% at approximately 100 °C, which was due to the evaporation of water from AZI (Figure 3). Therefore, it is necessary to ensure that the extrusion temperature of SD is below 200 °C to prevent the decomposition of AZI.

#### 3.1.3. Hot-Melt Extrusion

**Figure 4:**
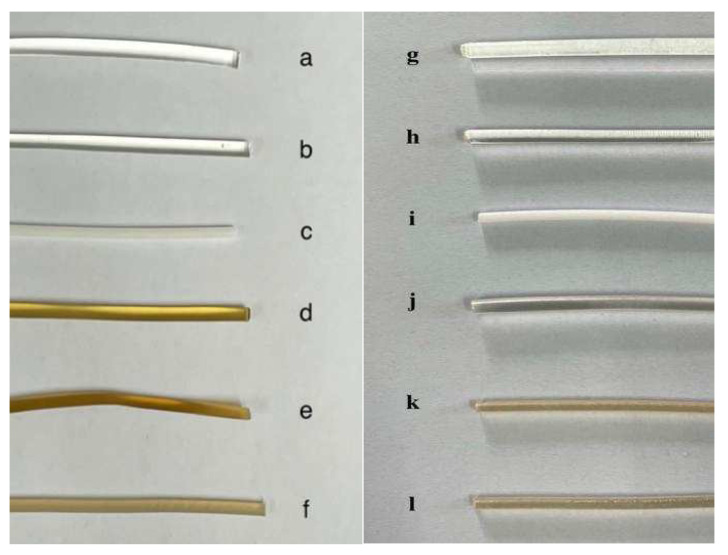
Solid dispersions of different polymers: AZI-Eudragit^®^ RL PO (**a**), AZI-Eudragit^®^ RS PO (**b**), AZI-GB (**c**), AZI-PVP K30 (**d**), AZI-HPC LXF (**e**), AZI-HPMC 15LV (**f**); Different Blank polymers: Eudragit^®^ RL PO (**g**), Eudragit^®^ RS PO (**h**), GB (**i**), PVP K30 (**j**), HPC LXF (**k**), HPMC 15LV (**l**).

The extrusion temperature is set by the properties of the polymer, and the extrusion speed is set to 100 rpm. The parameters of the extrusion process are shown in Table 6, and the appearance of the extrudates is shown in Figure 4.

#### 3.1.4. Taste-Masking Effect

**Figure 5:**
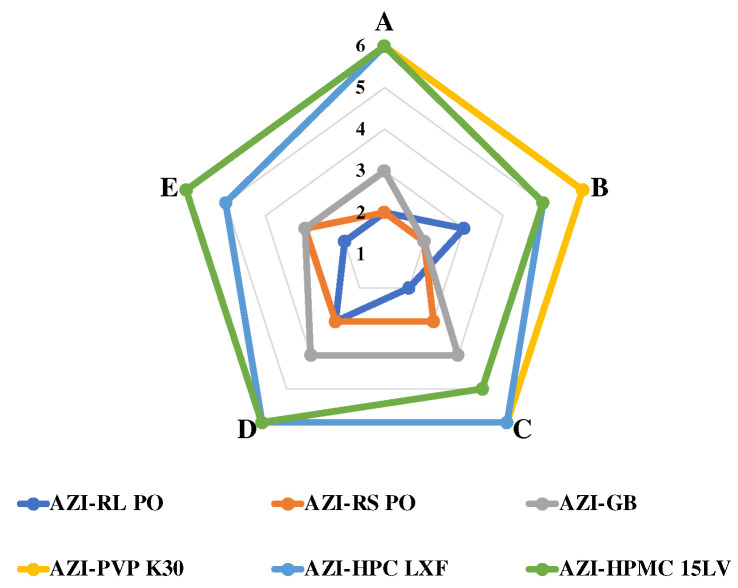
Evaluation of Bitterness of AZI-SD.

The ISEM method to evaluate the taste-masking effect of AZI-SD is shown in Figure 5. The polymers PVP K30, HPC LXF, and HPMC 15LV have poor taste-masking effects. Their bitterness scores are all above 5, so the bitterness is unacceptable. GB, Eudragit^®^ RL PO, and Eudragit^®^ RS PO have better taste masking effects. Their scores are within 2–3 points, an acceptable level of bitterness. Therefore, in order to mask the bitter taste of AZI, Eudragit^®^ RL PO, Eudragit^®^ RS PO and GB are chosen as polymers to prepare AZI-SD.

#### 3.1.5. Dissolution Testing

**Figure 6:**
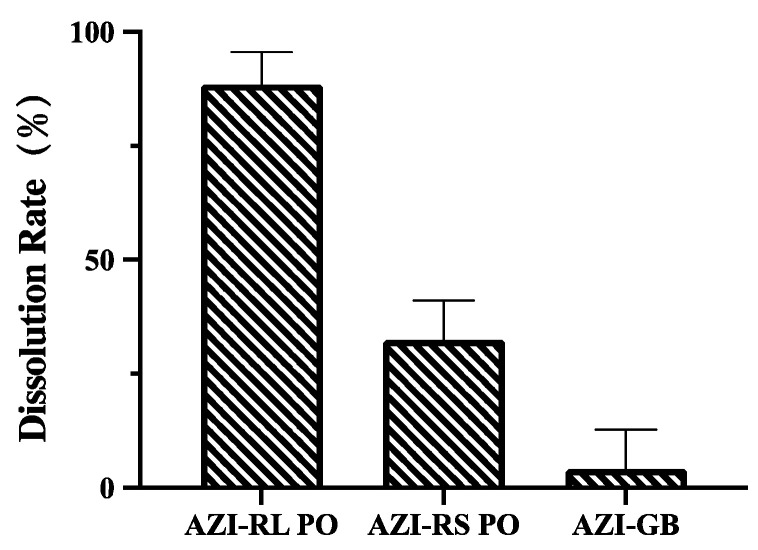
Different polymer dissolution results.

Dissolution rate experiments were performed based on polymers with good taste-masking effect. The results are shown in Figure 6. At 60 min, the dissolution rate of AZI-RL PO was 88.37%, AZI-RS PO was 32.41%, and AZI-GB dissolution rate was less than 5%. Based on the long half-life of AZI, the development of an immediate-release formulation is planned. Therefore, Eudragit^®^ RL PO was chosen as the polymer.

#### 3.1.6. Saturation Solubility Testing

**Figure 7:**
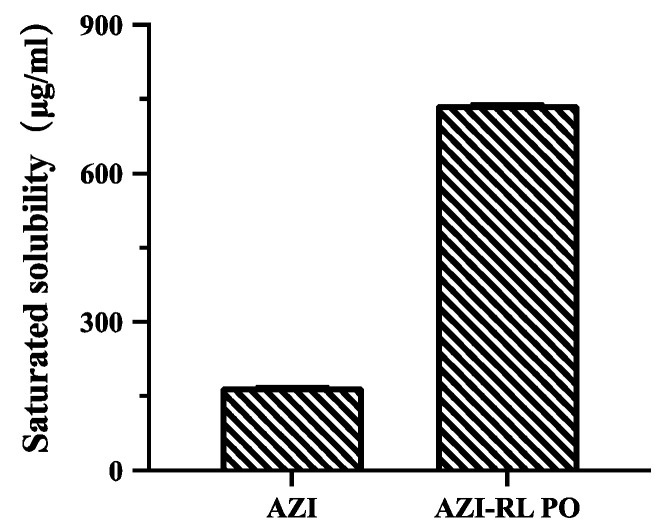
Saturated solubility of AZI and AZI-RL PO.

Eudragit^®^ RL PO was selected as a polymer for its taste masking effect and dissolution behavior, and its solubility was measured. The results of saturation solubility test are shown in Figure 7. The saturation solubility of AZI in water is only 170 μg/mL, while that of AZI-RL PO is 740 μg/mL, which is significantly better than AZI, indicating that the solubility of AZI could be improved by preparing AZI-RL PO with HME.

Therefore, Eudragit^®^ RL PO was selected as the polymer for the preparation of AZI-SD based on the extrusion process, taste masking effect, dissolution and solubility.

### 3.2. Analysis of DoE Results

According to the DoE results, there were significant differences in content, dissolution rate and extrudates diameter among batches. However, there was no significant difference in the appearance of the extrudates, which was considered as a narrow temperature setting range, so the appearance was not analyzed subsequently.

**Figure 8:**
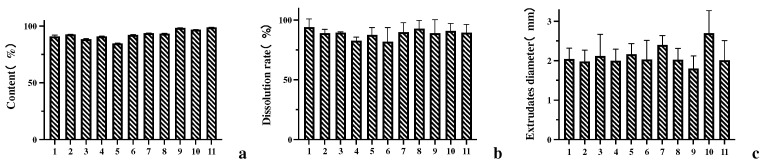
DOE test results: content (**a**); dissolution rate (**b**); extrudate diameter (**c**).

**Figure 9:**
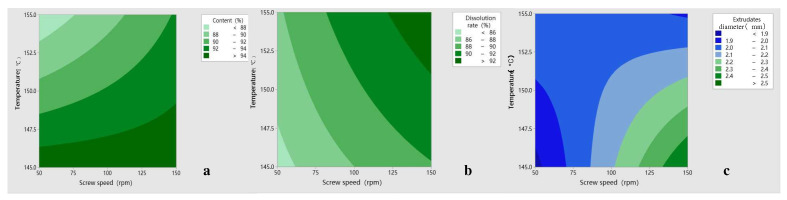
Contour map: content (**a**); dissolution rate (**b**); extrudate diameter (**c**).

From the results (Figure 8a and Figure 9a), it can be seen that the extrusion temperature is the main significant factor affecting the content, and the temperature directly affects whether the AZI degrades or not; also, the response to the content is negatively correlated. The effect of screw speed on the content was not significant, but shows a positive correlation. When the temperature is slightly higher (higher than 148 °C), the SD content at high screw speeds is higher than that at low screw speeds. The general trend is that the content of AZI-RL PO is higher as the temperature decreases and the screw speed increases.

The dissolution rate of 5 min was chosen as the response value for the experiment, and the results are shown Figure 8b and Figure 9b. It can be seen that temperature and screw speed are important factors affecting dissolution, with temperature having a greater effect on the dissolution of SD. Temperature and screw speed are both positively correlated with dissolution, and the dissolution rate of AZI-RL PO is relatively higher as the temperature increases and screw speed increases.

The diameter of the extrudates shows the smoothness of the extrusion process, which affects the size of the crushed particles in SD and the downstream processing of the preparation. The diameter of the extrusion die is 2 mm. In the experiment, it was found that under different parameters, the diameter of the extrudates differed. The results are shown in Figure 8c and Figure 9c. It can be seen that temperature is a significant factor affecting the diameter of the extrudates, and there is a certain interaction between temperature and screw speed. The response of temperature to the extrudates’ diameter is negatively correlated. As the speed increases and the temperature decreases, the diameter of the extrudates becomes thicker.

**Figure 10:**
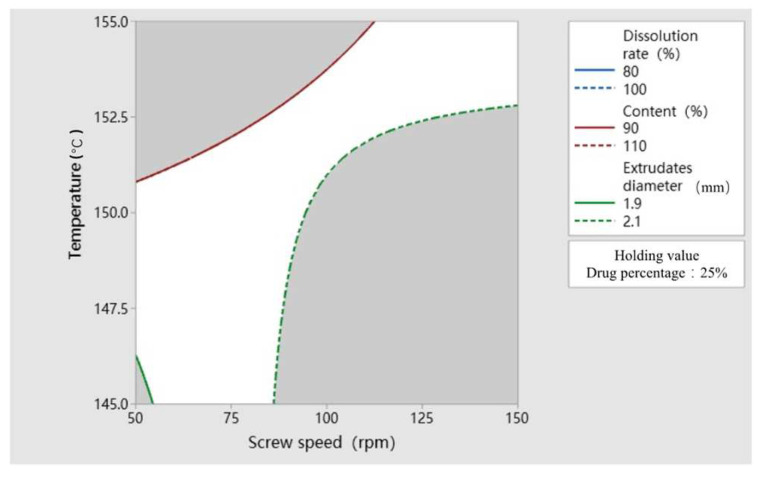
Design space for preparing AZI-RL PO by hot-melt extrusion.

The results of the above DoE study show that temperature and speed have an effect on multiple response values, and they have a certain interaction effect when affecting the response value. Therefore, the target range of each response variable is determined by combining the above influencing factors to establish the design space of HME parameters, as shown in the white area in Figure 10. Finally, it is determined that the extrusion parameters of HME to prepare AZI-RL PO are: temperature—150 °C; screw speed—75 rpm; and drug percentage—25%.

### 3.3. Physicochemical Evaluation

#### 3.3.1. Differential Scanning Calorimetry (DSC)

**Figure 11:**
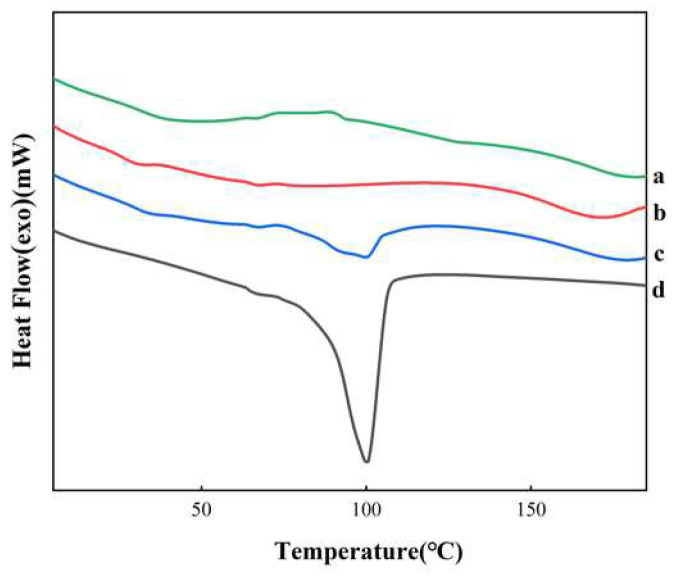
DSC thermal curve: AZI-RL PO (**a**); Eudragit^®^ RL PO (**b**); PM (**c**); AZI (**d**).

The DSC thermogram of the AZI and PM showed a melting peak at 125 °C (Figure 11) corresponding to the melting peak of the pure drug, while the AZI-RL PO did not have a melting peak of AZI, indicating the absence of crystalline AZI in the solid dispersion. This may be due to the conversion of crystalline AZI to the amorphous form.

#### 3.3.2. Powder X-ray Diffraction (PXRD)

**Figure 12:**
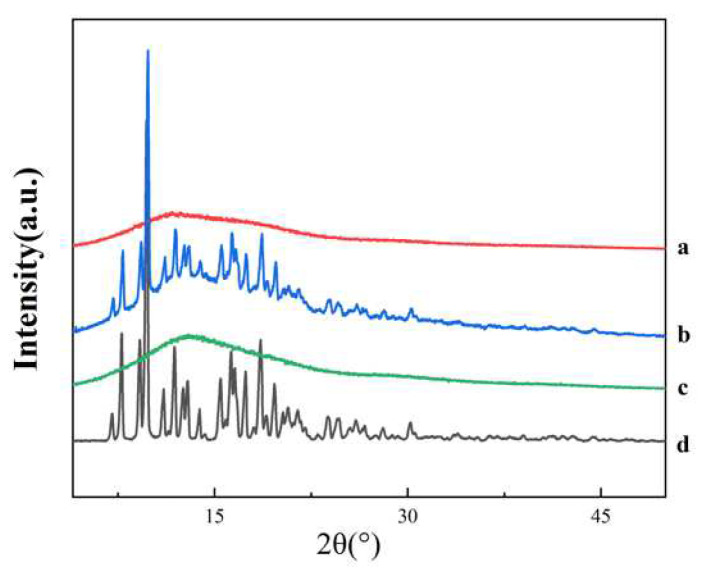
PXRD spectrum: AZI-RL PO (**a**); PM (**b**); Eudragit^®^ RL PO (**c**); AZI (**d**).

The PXRD pattern (Figure 12) showed a series of distinct crystal diffraction peaks for the pure AZI, indicating that it is a crystalline structure: the polymer of Eudragit^®^ RL PO appeared in an amorphous state; PM peaks correspond to pure crystalline AZI, but some of the peaks became weaker; and the crystal diffraction peaks of AZI-RL PO disappeared. Combined with the DSC spectrum results (Figure 8), it was concluded that the AZI in the extrudate was dispersed in Eudragit^®^ RL PO in an amorphous state, and formed a solid solution.

#### 3.3.3. Fourier Transform InfraRed (FTIR) Spectrophotometric Analysis

**Figure 13:**
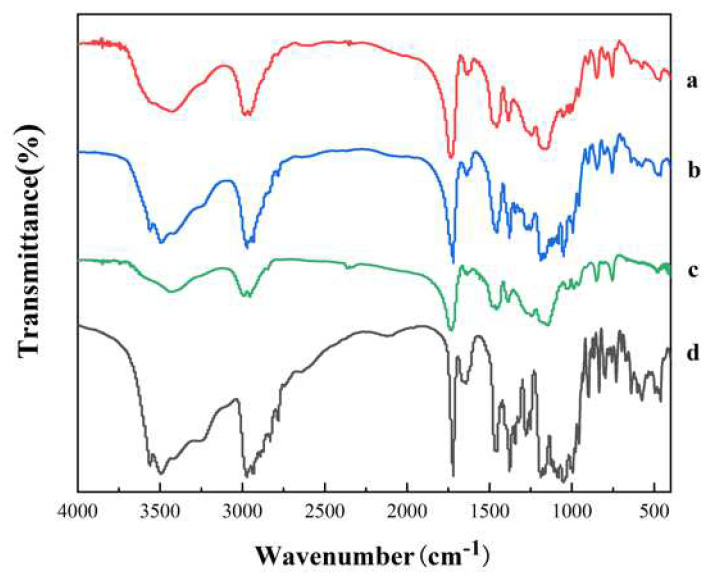
FTIR spectrum: AZI-RL PO (**a**), PM (**b**), Eudragit^®^ RL PO (**c**), AZI (**d**).

From the FTIR spectrum (Figure 13), it can be seen that AZI has an O–H stretching vibrational region at 3400 cm^−1^, a peak at 3494 cm^−1^ indicating the presence of “tightly bound” water, and a peak at 3560 cm^−1^, indicating the presence of two crystalline water in AZI [27,28], while the polymer does not have a stretching vibrational peak in this region. Meanwhile, for AZI-RL PO, the O–H stretching vibration peak ((3560 cm^−1^) disappeared and only a broad absorption peak (3419 cm^−1^), indicating the loss of AZI crystal water during the HME process and the formation of “loosely bound” water [27]. This shows a possible hydrogen bond interaction between AZI and Eudragit^®^ RL PO in solid dispersion prepared by HME.

In addition, the stretching vibration peaks of the carbonyl group (C=O) are also significantly different. AZI has a sharp and strong stretching vibration peak at 1720 cm^−1^; PM also has a carbonyl stretching vibration peak at 1720 cm^−1^. In AZI-RL PO, the peak wave number is 1734 cm^−1^, the peak is blue-shifted, and the peak intensity is reduced, indicating a hydrogen bond break between the water of crystallization and AZI.

## 4. Discussion

In HME technology, a large percentage of polymer is used in order to keep the amorphous AZI in a low saturation state and to form SS. Through the encapsulation of AZI by the polymer and the interaction between AZI and polymer (hydrogen bond), the contact between the drug and taste buds is reduced and the effect of taste masking is achieved [29,30,31]. At the same time, the taste-masking effect is also related to the polymer. The Eudragit^®^ series is a class of excipients widely used for taste masking in pharmaceutical formulations.

Mechanical energy combined with thermal energy provides energy for the API, but mechanical energy itself does not cause the degradation of the API, which is mainly influenced by thermal energy. Although TGA showed that AZI decomposes above 200 °C, preliminary polymer screening experiments found that when the extrusion temperature was 180 °C it caused partial degradation of AZI. It can be seen that temperature has a large effect on the content. DoE experiments show that temperature is negatively correlated with SD content, while screw speed has little effect on SD content. In the low temperature range, low or high screw speeds did not cause degradation of AZI. High screw speed was more effective than low screw speed when the temperature was higher than about 148 °C. This is because, under high screw speed conditions, the material stays in the barrel for a short time, enters and exits quickly and is not subjected to continuous heating.

AZI-SD was prepared with the aim of improving solubility and developing immediate-release formulations. The results showed that the higher the rotational speed, the faster the dissolution of SD in the same temperature range. With the same screw structure, increasing the rotational speed increases the mechanical shear energy, resulting in a more uniform dispersion of the drug in the amorphous state. Therefore, the dissolution rate will be increased. Similarly, at AZI thermal stability temperature, increasing the temperature will introduce more thermal energy and the dissolution rate of SD will be increased.

The diameter of the extrudates shows how smooth the extrusion process is. Since temperature mainly affects the melt state of the material, the higher the temperature, the better the flow of the material. The screw speed has less influence on the diameter of the extrudate. At low temperatures, the flowability is poor and the material tends to cause buildup, resulting in a thicker extrudate.

The results by DSC and PXRD showed that AZI was present in SD in an amorphous form. The interaction of the drug with the polymer was observed by FTIR. The changes in the spectra indicated the formation of hydrogen bonds between AZI and Eudragit^®^ RL PO. This indicates that the drug is dispersed in the polymer to form a solid solution [32].

**Figure 14:**
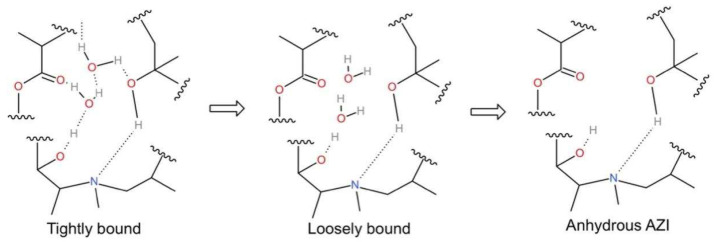
Schematic representation of disruption process of hydrogen bonds between AZI and water of crystallization.

Figure 14 shows the transition of AZI from crystalline to amorphous form. In the HME process, the hydrogen bonds between AZI and the water of the crystallization is broken and the “loosely attached” water is released outside the AZI lattice, which promotes the formation of hydrogen bonds between Eudragit^®^ RL PO and AZI, while AZI changes from crystalline to amorphous state [27,33].

The dissolution of AZI in the amorphous state requires lower energy than that in the crystalline state, and hydrogen bonds promote the solubility of AZI. Secondly, under the action of shear force, the particle size of the material keeps shrinking, which increases the specific surface area of the particles, increases the contact with the dissolution medium, and improves the wettability and agglomeration of the drug. As a result, the solubility of AZI-RL PO was greatly improved compared to that of crystalline AZI. The dissolution behavior of SD is also related to the nature of the polymer. Eudragit^®^ RL PO is a thermoplastic methacrylate copolymer in which the drug is released through hydrophilic pores formed by quaternary ammonium groups. It has a high content of quaternary ammonium groups, forming pores with a large diameter and high swelling and permeability, which allows a faster drug release [34,35].

## 5. Conclusions

In this study, Eudragit^®^ RL PO combined with HME was innovatively used to prepare AZI preparation, solving the problems of bitterness and solubility. It was shown that HME is a suitable technique for the preparation of AZI-SD. AZI was converted from crystalline to amorphous form and is interacting with the polymer Eudragit^®^ RL PO. The extrusion conditions play a crucial role in the feasibility of this technology, and by combining all the response indicators, the best response surface for the process prescription can be obtained. In conclusion, the availability of the Eudragit^®^ RL PO and HME brings a new research direction for poorly soluble and bitter-tasting drugs. It can lay a good foundation for the development of compliant pediatric preparations.

## Figures and Tables

**Table 1:** AZI solid dispersion bitterness grade.

Grade	Bitterness	Acceptability	Score
I	Not bitter	Acceptable	1
II	Slightly bitter		2
III	A little bitter		3
IV	Bitter	Unacceptable	4
V	More bitter		5
VI	Extremely bitter		6

**Table 2:** 2^3^ Full factor design of experiment.

Factor		Level		
		−1	0	1
A	Screw Speed (rpm)	150	100	50
B	Extrusion Temperature (℃)	145	150	155
C	Drug percentage (%)	20	25	30
**Response index**		**Acceptable interval**		
Y1	Appearance	Colorless transparent		
Y2	Content	90–110 %		
Y3	Dissolution rate	>80% *		
Y4	Extrudates diameter	1.9–2.1 mm		

* Five minutes of dissolution rate.

**Table 3:** Design planning of 2^3^ full factor experimental.

Run Sequence	Screw Speed (rpm)	Extrusion Temperature (°C)	Drug Percentage (%)
1	150	155	30
2	100	150	25
3	50	155	30
4	100	150	25
5	50	155	20
6	50	145	20
7	150	145	20
8	150	155	20
9	50	145	30
10	150	145	30
11	100	150	25

**Table 4:** HSP of Azithromycin.

Group	Number	Fdi (J^1/2^cm^3/2^/mol)	Fpi (J^1/2^cm^3/2^/mol)	Ehi (J/mol)	V (cm^−3^/mol)
CH3	14	420	0	0	33.5
CH2	5	270	0	0	16.1
CH	15	80	0	0	−1.0
C	4	70	0	0	−19.2
O	5	100	400	3000	3.8
OH	5	210	500	20,000	10.0
N	2	20	800	5000	−9.0
COO	1	390	490	7000	18.0
Σ	-	10,690	3,570,100	132,000	526.7
δ	-	20.30	3.59	15.83	-

**Table 5:** HSPs of Polymers.

Drug/Polymer	Δ (MPa^0.5^)	Δδ (MPa^0.5^)
AZI	25.99	-
Eudragit^®^ RL PO	20.27 [25]	5.72
Eudragit^®^ RS PO	20.37 [25]	5.62
GB	20.15	5.84
PVP K30	22.5	3.49
HPC LXF	21.27 [21]	4.72
HPMC 15LV	22.40	3.59
Kollicoat^®^ Protect	34.0 [26]	8.01

**Table 6:** Different Carrier extrusion parameters.

Polymer	Drug Content (%)	Temperature (°C)	Torque (%)	Appearance
Eudragit^®^ RL PO	50	150	40	Colorless transparent
Eudragit^®^ RS PO	50	143	40	Colorless transparent
GB	50	73	10	White opaque
PVP K30	50	185	10	Brown transparent
HPC LXF	50	180	30	Brown transparent
HPMC 15LV	50	145	45	Light yellow transparent
Eudragit^®^ RL PO	50	150	40	Colorless transparent
Eudragit^®^ RS PO	50	143	40	Colorless transparent

## Data Availability

Not applicable.

## References

[1] Adams L.V., Craig S.R., Mmbaga E.J., Naburi H., Lahey T., Nutt C.T., Kisenge R., Noel G.J., Spielberg S.P. (2013). Children’s Medicines in Tanzania: A National Survey of Administration Practices and Preferences. PLoS ONE.

[2] Rodriguez W., Selen A., Avant D., Chaurasia C., Crescenzi T., Gieser G., Di Giacinto J., Huang S.-M., Lee P., Mathis L. (2008). Improving Pediatric Dosing Through Pediatric Initiatives: What We Have Learned. Pediatrics.

[3] Thabet Y., Klingmann V., Breitkreutz J. (2018). Drug Formulations: Standards and Novel Strategies for Drug Administration in Pediatrics. J. Clin. Pharmacol..

[4] Wang Z., Han X., Chen R., Li J., Gao J., Zhang H., Liu N., Gao X., Zheng A. (2021). Innovative color jet 3D printing of levetiracetam personalized paediatric preparations. Asian J. Pharm. Sci..

[5] Wang Z., Li J., Hong X., Han X., Liu B., Li X., Zhang H., Gao J., Liu N., Gao X. (2021). Taste Masking Study Based on an Electronic Tongue: The Formulation Design of 3D Printed Levetiracetam Instant-Dissolving Tablets. Pharm. Res..

[6] Smith C., Egunsola O., Choonara I., Kotecha S., Jacqz-Aigrain E., Sammons H. (2015). Use and safety of azithromycin in neonates: A systematic review. BMJ Open.

[7] Amin F., Khan S., Shah S.M.H., Rahim H., Hussain Z., Sohail M., Ullah R., Alsaid M.S., Shahat A.A. (2018). A new strategy for taste masking of azithromycin antibiotic: Development, characterization, and evaluation of azithromycin titanium nanohybrid for masking of bitter taste using physisorption and panel testing studies. Drug Des. Dev. Ther..

[8] Firth A., Prathapan P. (2020). Azithromycin: The First Broad-spectrum Therapeutic. Eur. J. Med. Chem..

[9] Abou Assi R., Abdulbaqi I.M., Seok Ming T., Siok Yee C., Wahab H.A., Asif S.M., Darwis Y. (2020). Liquid and Solid Self-Emulsifying Drug Delivery Systems (SEDDs) as Carriers for the Oral Delivery of Azithromycin: Optimization, In Vitro Characterization and Stability Assessment. Pharmaceutics.

[10] Rajesh A.M., Popat K.M. (2016). Taste masking of azithromycin by resin complex and sustained release through interpenetrating polymer network with functionalized biopolymers. Drug Dev. Ind. Pharm..

[11] Huang R., Zhang Y., Wang T., Shen L., Zhang Z., Wang Y., Quan D. (2018). Creation of an assessment system for measuring the bitterness of azithromycin-containing reverse micelles. Asian J. Pharm. Sci..

[12] Mishra D.K., Dhote V., Bhargava A., Jain D.K., Mishra P.K. (2015). Amorphous solid dispersion technique for improved drug delivery: Basics to clinical applications. Drug Deliv. Transl. Res..

[13] Potta S.G., Minemi S., Nukala R.K., Peinado C., Lamprou D., Urquhart A., Douroumis D. (2010). Development of Solid Lipid Nanoparticles for Enhanced Solubility of Poorly Soluble Drugs. J. Biomed. Nanotechnol..

[14] Cid A.G., Simonazzi A., Palma S.D., Bermúdez J.M. (2019). Solid dispersion technology as a strategy to improve the bioavailability of poorly soluble drugs. Ther. Deliv..

[15] Baghel S., Cathcart H., O’Reilly N.J. (2016). Polymeric Amorphous Solid Dispersions: A Review of Amorphization, Crystallization, Stabilization, Solid-State Characterization, and Aqueous Solubilization of Biopharmaceutical Classification System Class II Drugs. J. Pharm. Sci..

[16] Budhwar V., Kaushik D. (2020). An Overview on Recent Patents and Technologies on Solid Dispersion. Recent Pat. Drug Deliv. Formul..

[17] Wilson M., Williams M.A., Jones D.S., Andrews G.P. (2012). Hot-melt extrusion technology and pharmaceutical application. Ther. Deliv..

[18] Thakkar R., Thakkar R., Pillai A., Ashour E.A., Repka M.A. (2020). Systematic screening of pharmaceutical polymers for hot melt extrusion processing: A comprehensive review. Int. J. Pharm..

[19] Patil H., Tiwari R.V., Repka M.A. (2016). Hot-Melt Extrusion: From Theory to Application in Pharmaceutical Formulation. AAPS PharmSciTech.

[20] Lang B., McGinity J.W., Williams R.O. (2014). Hot-melt extrusion—Basic principles and pharmaceutical applications. Drug Dev. Ind. Pharm..

[21] Ponnammal P., Kanaujia P., Yani Y., Ng W.K., Tan R.B.H. (2018). Orally Disintegrating Tablets Containing Melt Extruded Amorphous Solid Dispersion of Tacrolimus for Dissolution Enhancement. Pharmaceutics.

[22] Sarode A.L., Sandhu H., Shah N., Malick W., Zia H. (2013). Hot melt extrusion (HME) for amorphous solid dispersions: Predictive tools for processing and impact of drug–polymer interactions on supersaturation. Eur. J. Pharm. Sci..

[23] Forster A., Hempenstall J., Tucker I., Rades T. (2001). Selection of excipients for melt extrusion with two poorly water-soluble drugs by solubility parameter calculation and thermal analysis. Int. J. Pharm..

[24] Mathieu D. (2018). Pencil and Paper Estimation of Hansen Solubility Parameters. ACS Omega.

[25] Quinten T., Andrews G.P., de Beer T., Saerens L., Bouquet W., Jones D.S., Hornsby P., Remon J.P., Vervaet C. (2012). Preparation and Evaluation of Sustained-Release Matrix Tablets Based on Metoprolol and an Acrylic Carrier Using Injection Moulding. AAPS PharmSciTech.

[26] Kolter K., Karl M., Gryczke A. (2012). Hot Melt Extrusion with Basf Pharma Polymers.

[27] Li X., Peng H., Tian B., Gou J., Yao Q., Tao X., He H., Zhang Y., Tang X., Cai C. (2015). Preparation and characterization of azithromycin—Aerosil 200 solid dispersions with enhanced physical stability. Int. J. Pharm..

[28] Sundaramurthi P., Suryanarayanan R. (2014). Azithromycin Hydrates—Implications of Processing-Induced Phase Transformations. J. Pharm. Sci..

[29] Maniruzzaman M., Boateng J., Bonnefille M., Aranyos A., Mitchell J., Douroumis D. (2012). Taste masking of paracetamol by hot-melt extrusion: An in vitro and in vivo evaluation. Eur. J. Pharm. Biopharm..

[30] Douroumis D. (2007). Practical approaches of taste masking technologies in oral solid forms. Expert Opin. Drug Deliv..

[31] Douroumis D. (2011). Orally disintegrating dosage forms and taste-masking technologies. Expert Opin. Drug Deliv..

[32] Kadajji V.G., Betageri G.V. (2011). Water Soluble Polymers for Pharmaceutical Applications. Polymers.

[33] Neglur R., Hosten E., Aucamp M., Liebenberg W., Grooff D. (2018). Water and the relationship to the crystal structure stability of azithromycin. J. Therm. Anal. Calorim..

[34] Thakral S., Thakral N.K., Majumdar D.K. (2012). Eudragit^®^: A technology evaluation. Expert Opin. Drug Deliv..

[35] dos Santos J., da Silva G.S., Velho M.C., Beck R.C.R. (2021). Eudragit^®^: A Versatile Family of Polymers for Hot Melt Extrusion and 3D Printing Processes in Pharmaceutics. Pharmaceutics.

